# Medicinal Treatment of Elderly Psoriasis Patients before and after Entering a Nursing Home

**DOI:** 10.3390/healthcare10091730

**Published:** 2022-09-08

**Authors:** Jana Petersen, Claudia Garbe, Sandra Wolf, Brigitte Stephan, Matthias Augustin, Kristina Hagenström

**Affiliations:** Institute for Health Services Research in Dermatology and Nursing (IVDP), University Medical Center Hamburg-Eppendorf (UKE), 20246 Hamburg, Germany

**Keywords:** nursing home residents, statutory health insurance, health services research, drug supply, Germany, skin disease

## Abstract

Psoriasis (PS) is a chronic inflammatory skin disease, and it increasingly appears also in the elderly population. There is a rising interest in drug therapy for PS, especially for people receiving care in nursing homes (NH). Which PS-related drugs are prescribed in the time before nursing home admission (NHA), and to what extent does the supply of drugs change after NHA? Which specialties prescribe PS-related drugs? Statutory health insurance data were examined for people with PS, aged ≥ 65 years, who were newly admitted to a NH in the period 2011–2014 and observed for one year before and after NHA. Changes in prescription prevalence (pre-post comparison) were examined for significant differences. Prescriptions of PS-relevant drugs were measured by defined daily dose and stratified according to the prescribing specialist group. The analysis included 718 insured persons with PS (76.2% female, mean age 83.3 years). Systemic therapeutics played a minor role (pre: 2.6% vs. post: 2.1%) in drug therapy. Topical steroids had a high share of about 40% in the pre–post comparison. Overall, the proportion of people with PS who received treatment remained at a comparable level before and after NHA. A structured assessment of the skin is crucial, specifically in people with cognitive impairment.

## 1. Introduction

Psoriasis is a chronic inflammatory skin disease characterized by skin and joint manifestations. Psoriasis is associated with a high degree of morbidity and decreased quality of life [1,2,3]. This may particularly affect people of older age [4]. According to today’s view, psoriasis is a genetically co-conditioned inflammatory disease in which the immune system is overactive and significantly involved in triggering the disease. The inflammatory signals of the immune cells activate the epidermal cells, which then begin to proliferate, accelerating the zytokine pathways. In some of those affected, typical triggers of psoriasis can be found, including, for example, infections with streptococci, certain drugs such as beta blockers or antimalarials, mechanical stimuli and stress [5,6,7,8].

In most people, psoriasis appears on the extensor sides of the elbows or knees, on the low back and on the hairy head. In some cases, psoriasis extends over large parts of the body surface. In many cases, the nails are also affected [9]. The individual inflammatory foci are sharply delimited and often scaly. In addition, a large proportion of those affected develop an annoying itching or skin pain at these lesions. A smaller proportion of about 10% of those affected have a different skin appearance—they tend to have pustules rather than scaling, or the inflammation affects bends and folds of the skin [10].

Due to the chronic nature of psoriasis and increasing life expectancy, psoriasis significantly affects the elderly population. Hence, the management of psoriasis in the aged population has grown into an important healthcare problem [11,12]. Demographic studies predict that by 2025, up to 25% of the U.S. population will be elderly (≥ 65 year), and for Europe, 34% is anticipated for 2050 [12]. Today, studies implicate that about 10% of psoriasis patients are elderly [13]. Determining the best individual treatment of psoriasis is challenging due to multimorbid patients who are usually treated with various drugs that can cause adverse drug reactions and drug interaction. Age-related changes in pharmacokinetics and pharmacodynamics contribute to this problem. However, conditions limiting the patient’s ability to follow treatment instructions, for example, reduced vision and hearing, also play an increasing role in clinical care [14]. In general, age-related changes in the skin occur: skin becomes drier, reducing its resistance to environmental influences and having a diminished regeneration potential [15]. Another problem is the lack of tailored treatment approaches for the needs of chronically multimorbid and elderly patients. This problem is specifically applicable to psoriasis, as it is not realised as a typical disease of advanced age. Regularly, patients over 65 years of age are not included in randomised clinical trials in pharmacotherapy. Consequently, innovations in drug therapy often do not reach older people and thus have only limited benefit for this population group [16].

A number of treatment options are available for psoriasis: In addition to a basic therapy providing nourishing and moisturising effects for the skin as well as preparations with urea and salicylic acid, topical and systemic treatment options as well as phototherapy are available, and standard regimens are established [8]. New treatment approaches have been developed, especially for long-term treatment with topical therapies for mild–moderate disease conditions, which now also need to be examined in comparison with conventional therapies [17].

For patients with moderate to severe psoriasis, biological and nonbiological systemic therapeutics are available. The approval of biologics in particular has revolutionised the treatment of psoriasis. The variety of options offers an individual approach for patients. Psychosocial therapy can be a supporting stimulus and be part of the therapy plan. The Psoriasis Area and Severity Index (PASI) is commonly used as a measure for the severity of psoriasis and treatment effectiveness. The PASI rates the clinical appearance of the disease with redness, scaling and thickness of the plaques and includes the extent of the affected skin area. A PASI of 10 or more (scale in the range 0–72) indicates moderate to severe psoriasis [8].

Little is known about the care of older people with psoriasis in the nursing home (NH) setting or how care changes as a result of entering a NH. The aim of this study was to investigate which psoriasis-relevant drugs are used in the year before vs. the year after nursing home admission and whether the spectrum of drugs changed.

## 2. Material and Methods

### 2.1. Database and Study Cohort

The present analysis is based on data of the DAK-Gesundheit (DAK-G), a nationwide-operating health insurance company. With about six million members, the DAK-G is one of the largest health insurance funds in Germany. The claims (routine) data covers a 40% representative sample (2.4 million) of all insured people of the DAK-G on 31 December 2010 and is available for this population until 31 December 2015. The data contains all billing-relevant information from the outpatient and inpatient sector, including outpatient-prescribed drugs (based on the Anatomical Therapeutic Chemical (ATC) Classification System), coded diagnoses (according to the German modification of the International Classification of Diseases (ICD-10-GM) and outpatient contacts with physicians at a quarterly level. In Germany, outpatient diagnoses are coded on a quarterly basis. Every statutory health insurance fund also includes a statutory long-term care insurance fund. The data of the German long-term care insurance (Gesetzliche Pflegeversicherung) contains information on a daily basis about the date of nursing home admittance (NHA) as well as the degree of need of care (ranging from care level 1, considerable need of care, to care level 3, most heavily care dependent). For detailed information on the German healthcare system as well as the system of long-term care insurance, refer to Busse and Blümel [18].

Our study included all insured people of the DAK-G sample aged 65 years and older, who were newly admitted to a NH between 1 January 2011 and 31 December 2014, with a subsequent insurance period of at least one day each in at least four of the following four quarters. Additionally, the study population received at least one confirmed ambulatory (outpatient) diagnosis of psoriasis or at least one main diagnosis of psoriasis on discharge of inpatient care in the year before NHA (Table 1). The four quarters before entering the nursing home (including the quarter of NHA) were compared with four subsequent quarters after NHA. To ensure a comparable time at risk in both time periods, the study population of NH residents was insured at least one day in each of the four quarters after NHA.

### 2.2. Psoriasis

The ICD-10 codes in Table 1 were used to identify patients with psoriasis.

### 2.3. Severe Psoriasis

For identifying patients with a moderate to severe form of psoriasis, the following criteria were used:

One inpatient main diagnosis of psoriasis OR at least one prescription for the followingdrugs based on the ATC information within the corresponding year (Table 2).

If an insured person does not meet the above criteria for systemic therapy but has the diagnosis of psoriasis any time point, they are classified as mildly affected.

Specific comorbidities that play an important role in psoriasis, in dermatology as well as in geriatric care, were evaluated. The following diagnoses were examined: diabetes, obesity, hypertension, ischemic heart disease, osteoporosis, pruritus, cataract, depression and dementia [19,20]. Diagnoses were considered if they were coded at least once as a confirmed diagnosis in the outpatient setting in the year before or after NHA. The comorbidities were used to describe the burden of disease.

### 2.4. Prescription Prevalence

Prescription prevalence was defined as at least one prescription for the psoriasis-relevant agents: Table 3 shows systemic therapeutics, and Table 4 lists topical therapeutics that were included as psoriasis-relevant drugs based on ATC information.

In addition, we examined the quantity of drug groups, as measured in defined daily doses (DDD), stratified by prescribing specialist group.

To analyse prescription patterns and detect inadequate treatment, we examined the extent to which a reduction in biologic or nonbiologic systemic therapeutics is associated with an increase in systemic steroids. Additionally, we analysed the association between reduction in systemic therapeutics and zero prescriptions for topical steroids. Furthermore, we examined the extent to which a diagnosis of psoriatic arthritis is concomitant with systemic steroid therapy. We were also interested in whether a prescription for calcipotriol was prescribed in more than two consecutive quarters and how often combination preparations were prescribed.

In addition, to represent the morbidity and disease spectrum of the examined population, the top five most frequently prescribed agents were evaluated.

The study was conducted according to the Declaration of Helsinki. We considered the STROSA statement and the criteria of a national good practice guideline. According to the Good Practice of Secondary Data Analysis, a national guideline for the use of administrative databases, no approval of an ethical committee was required [22].

### 2.5. Statistical Analyses

First, descriptive methods were used to examine the prescription prevalence of general as well as psoriasis-related drugs and to compare the results in the year before to after NHA. McNemar´s test was used to assess differences between the two time periods. Analyses were stratified by age group (65–74 years, 75–84 years, 85+ years), care levels (0/1, 2, 3) and sex (male, female). A paired samples t-test was used to compare the drug quantity (DDD). Psoriasis-relevant drugs (DDD) were evaluated stratified by prescribing specialist group. Level of significance was set at *p* = 0.05. We conducted all statistical analysis using SAS for Windows, Version 9.4 (SAS Institute Inc., Cary, NC, USA).

## 3. Results

### 3.1. Baseline

Between 2011 and 2014, about 41,000 insured persons were newly admitted to a nursing home. Of these persons, 718 had at least one psoriasis diagnosis before NHA and could be observed for the period for one year before or one year after NHA. Mean age of included people was 83 (SD = 7.5) years, and two-thirds were female. The degree of need for care increased noticeably in the year following NHA: While about 4% of the study population were in the highest level of need of care before NHA, the proportion increased to around 11% in the following year. Almost all psoriasis patients were identified to suffer from mild psoriasis. Overall, a large number of different diseases was identified within the study population. The proportion of patients with the selective comorbidities (Table 5) after NHA decreased, apart from patients with pruritus and dementia, although these are also chronic illnesses (hypertension: prior NHA 85.2% vs. after NHA 79.5%).

### 3.2. Prescription Prevalence

Initially, for showing the spectrum of disease and the degree of morbidity, we examined all prescribed drugs. The analysis of the most frequent prescriptions before and after NHA showed that metamizole (pain medication) is the most frequently prescribed drug, followed by a diuretic agent, antihypertensives and a proton pump inhibitor (Table 6). These drugs also remained in the top five of most prescribed drugs after NHA, albeit in a slightly different ranking. One half of psoriasis patients received at least one prescription for a beta blocker (ATC code: C07A) with a significant reduction to 46.9% after NHA (*p* = 0.0052, data not shown).

### 3.3. Psoriasis Relevant Therapy

Nearly 100% of the insured population with psoriasis received at least one drug prescription within both time periods (Table 7). The number of prescriptions increased significantly from about 27,900 to about 33,300 after NHA (*p* < 0.0001). People with psoriasis received different active ingredients with a mean of 14.3 (SD = 6.5) before NHA and 13.2 (SD = 5.6) after NHA, respectively. About half of these patients received at least one psoriasis-relevant therapy (Table 7). There were no significant differences in the proportion of patients with psoriasis diagnosis with at least one psoriasis-relevant therapy in the before–after comparison (stratified by age, sex and care level). If the diagnosis of psoriasis was made by a dermatologist (prior to NHA n = 224; after NHA n = 134), the proportion of insured people who received at least one psoriasis-related therapy was 74% and 79%, respectively (data not shown). A stratified analysis of 45 and 31 persons with psoriasis and with at least one psoriatic arthritis diagnosis (ICD L40.5) showed that 53.2% and 41.9%, respectively, received at least one prescription for systemic therapy (at least one prescription for biological, nonbiological systemic therapeutics or systemic steroids).

### 3.4. Systemic Therapies in Psoriasis

Only a small proportion of patients was treated with systemic agents (2.6% vs. 2.1%; *p* = 0.2482). The following describes the extent to which a reduction in biologic or nonbiologic systemic therapeutics is associated with an increase in systemic steroids. In the present analysis, a reduction in system therapeutics after NHA affected only 8 persons with psoriasis (data not shown). Of these, no person received a new steroid prescription after NHA. A total of 19 individuals had a prescription for a systemic therapeutic before NHA. DDDs decreased in 12 of these persons with psoriasis after NHA. In 10 of 12 individuals (83.3%), therapy was not adjusted after NHA. A new prescription for a topical steroid occurred in 2 persons with psoriasis (data not shown). The proportion of patients with psoriasis with at least one prescription with systemic steroids decreased significantly from 17.5% before NHA to 13.8% after NHA (*p* = 0.0097). This applies primarily to the middle-aged and especially to women (Table 8).

### 3.5. Topical Therapy

**Figure 1:**
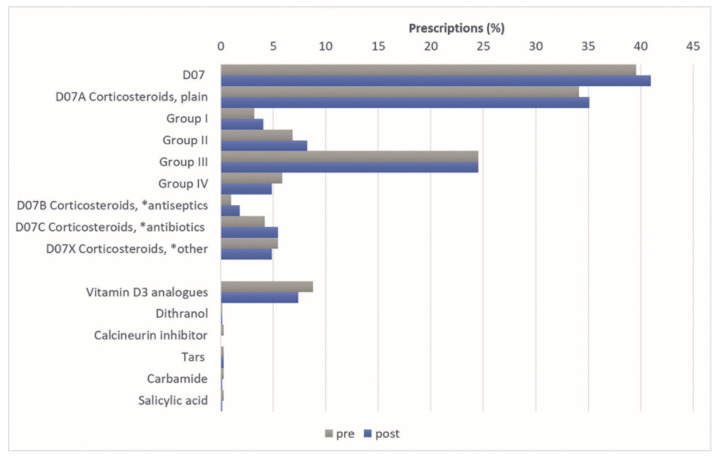
Topical therapy in psoriasis patients one year prior to one year after nursing home admission (N = 718); at least one prescription. * in combination with.

About 40% of the study population received at least one prescription for a topical steroid in both years (39.6% vs. 40.9%; Figure 1). In particular, plain steroids (ATC D07A) were prescribed in 34.1% vs. 35.1%. There were no significant differences prior vs. after NHA stratified by sex, age and care level. None of the other relevant topical treatment options appeared to be significant in the present analysis. Only vitamin D3 analogues were prescribed more frequently than other topical treatment options (8.8%, 6.3%, respectively; Figure 1). We analysed the prescription of calcipatriol in depth (data not shown). Of the 63 insured patients with calcipotriol prescriptions, 31.7% received a monopreparation prescription. Prior to NHA, a total of 57.1% received combination preparations, and the remaining patients received both types of prescriptions. Overall, 27 persons with psoriasis received monopreparation prescriptions and 25.9% (n = 7) of these in consecutive quarters. In the period after NHA, the value for monopreparation prescriptions remained at a comparable level of 32.1%. Of those persons with monopreparation prescriptions (n = 25), 36% (n = 9) received these prescriptions in at least two consecutive quarters. Basic therapeutics were prescribed less often.

### 3.6. Prescribing Specialist Group

**Figure 2:**
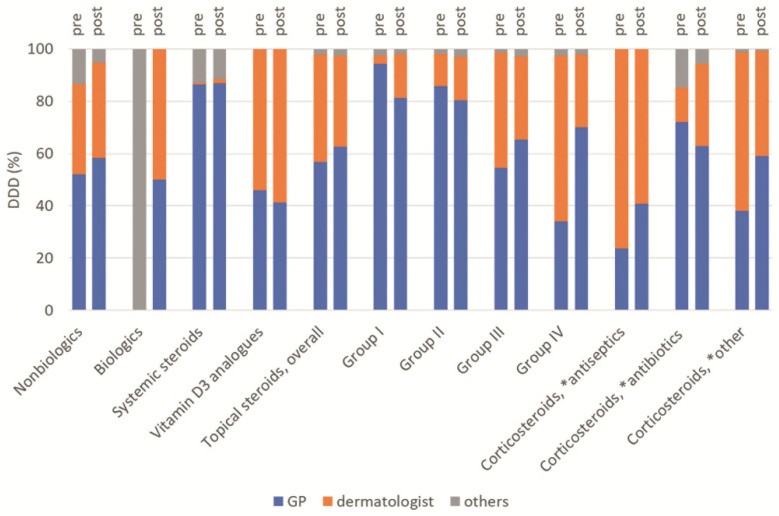
Proportion of defined daily dose (DDD) of the most prescribed psoriasis-relevant drugs by prescribing specialist group in pre–post comparison (N = 718 patients). GP, general practitioner; * in combination with.

Our analyses included biological or nonbiological systemic therapeutics as well as the frequently prescribed topical corticoids and vitamin D3 analogues. Due to the central role of the general practitioner (GP) in the care of people in NHs, drug therapy is primarily prescribed by the GP. The analysis of the quantity of drugs prescribed shows that mainly dermatologists prescribed more potent topical corticoids class III and IV. The same applies to steroids with antiseptics and other combinations (Figure 2).

## 4. Discussion

The world’s population is undergoing demographic change. In Germany, one in three people will be at least 65 years old by the year 2060 [24]. Demographic ageing means that age-associated chronic diseases will become more frequent and challenge the structures of health and nursing care [25]. Due to the demographic change, there will be a decline in informal caregivers, especially family members. Consequently, the importance of care in nursing facilities will increase in the future. People who are about to enter a NH or who live in a NH are often characterised by multimorbidity and complex health problems. Skin diseases play a significant role in this setting and can thus significantly influence the quality of life [2,26]. Due to the chronic character of psoriasis, adequate care and treatment will be meaningful against the background of demographic change. Psoriasis is not recognised as a relevant disease of old age and is therefore not sufficiently considered in therapy recommendations.

To date, there have been few studies on the care situation of older people with psoriasis and drug supply in this target population [11,27,28]. The current study refers to the specific context of care in the year before and after NHA. This phase is of particular importance for the individual, but also for the healthcare system. There is a risk that important information will be lost during the transition to a NH, which emphasises the need for information on the care situation and drug supply in this phase of life.

### 4.1. Drug Supply of Patients with Psoriasis

Almost all nursing home residents received at least one prescription of drug therapy before and after NHA. On average, they received around 13 and 14 different active ingredients, respectively. Metamizole appeared to be the most frequently prescribed drug. This finding is confirmed by previous studies on the basis of health insurance data—regardless of skin diseases [29,30]. The variety of agents analysed here, such as those to treat cardiovascular disease, reflect the typical disease spectrum of the population living in nursing homes. Psoriasis-related agents do not appear at this point.

### 4.2. Treatment of Psoriasis

Patient and care giver should define a common treatment goal at the beginning of each treatment [8]. While the therapeutic goal for young patients is defined concisely as freedom from appearance, little research has been conducted on what older patients hope to gain from treatment. Consequently, the first step should define an individual therapy goal. Overall, about 53% of the patients received psoriasis-relevant therapies before and after NHA. Therefore, it appears that the moment of NHA does not impact the treatment regimen noticeably. A detailed analysis of drug supply is described below.

### 4.3. Systemic Therapies

Our analyses show that systemic therapies play a minor role in the care of people with psoriasis as only about 2% received at least one prescription. Above all, biologics were almost not prescribed at all. In a cross-sectional study based on Medicare data from the U.S., in 2011, 27.3% of persons with psoriasis over 65 years were diagnosed with moderate to severe psoriasis, and 14.3% received systemic oral therapy [28]. A total of 10% of patients with psoriasis aged 65 and older received biologics. Based on these billing data, 27.3% of the patient population were classified as having moderate to severe psoriasis and were thus more severely affected than the population in our study (approx. 3%). The disease stage in the study by Takeshita and colleagues was defined by the presence of prescriptions for biologic or nonbiologic systemic therapeutics or phototherapy and is similar to our approach. Moreover, we refer to a specific population of nursing home residents, which must be considered a much frailer collective compared to the overall elderly population. Takeshita and colleagues also emphasised the difficulty to assess appropriate care on the basis of lacking clinical parameters in health insurance data. In addition, systemic therapeutics must always be prescribed under consideration of comorbidities, co-medications and age-related changes in pharmacokinetics and dynamics. According to studies, biologics can be a valuable treatment option in the elderly patient population, but data on systemic therapeutics in older psoriasis patients are scarce because the elderly are underrepresented in studies [16,31,32]. Di Caprio and colleagues recently reviewed the use of topical as well as systemic therapies and examined safety outcomes in older population [33]. The current guideline provides guidance on prescribing medications in the context of comorbidity, which is particularly relevant to the elderly. Age-related changes in drug administration are pointed out [8].

Our analyses show that biological or nonbiological systemic therapeutics decreased without an increase in topical steroids. Comparing treatment recommendations for psoriasis vulgaris (current S3 guideline) with the treatment situation reflected by health insurance data may be considered as a hint for inadequate care. Of the 19 total individuals with a prescription for a systemic therapeutic, 12 individuals received a reduced number of DDDs after NHA. These low patient numbers and current results point out that agent discontinuation should be exercised with caution, and other treatment options should be continued to reduce disease activity. Patients should be monitored closely to prevent exacerbation. Therefore, structured patient observation and documentation is essential. However, it must be emphasised that there is currently no nursing expert standard for skin and skincare, whereas it is well established for, e.g., malnutrition or decubitus prophylaxis. Structured documentation and handover would be of great importance, especially in the NH setting or during the transition to a NH.

Systemic steroids are used in about one-fifth of those with the disease. This proportion is significantly reduced and mainly affects women. Systemic steroids have been approved in a broad spectrum of indications. They are not included in guideline recommendations for the treatment of psoriasis vulgaris. Our results may hint at an inadequate supply, as biologic or nonbiologic systemic therapeutics decreased without an increase in topical steroids. Overall, biological or nonbiological agents play a minor role in the treatment situation of older psoriasis patients. A total of eight individuals experienced a reduction, but no one received a new prescription for steroids.

About half of our study population received at least one prescription for a beta blocker. This is not surprising in a population that typically has a high incidence of cardiovascular disease. However, in the context of psoriasis treatment, the use of beta blockers can trigger disease activity [34]. This must be considered when prescribing these agents.

### 4.4. Topical Therapies

Topical medications are of particular importance in the treatment of psoriasis, as systemic agents must be administered with caution in the context of polymedication and multimorbidity. Hence, topical agents are often prescribed as first-line therapy in elderly psoriasis patients. Topicals are to be used in the younger age group primarily for the treatment of the mild-to-moderate psoriasis [27]. Topical therapy plays a major role in the present analyses, notably topical steroids. The high prescription prevalence of topical therapy that is shown in our analyses is confirmed by Takeshita et al., who also examined elderly psoriasis patients. A total of 76.6% patients with psoriasis received topical therapy and most frequently topical steroids [28]. In our study, about 40% of patients received one of the relevant topical steroid prescriptions before and after NHA. Plain steroids (D07A) in particular were prescribed frequently (Figure 1). The analyses of prescriptions by the specialist group also show that potent steroids are prescribed primarily by dermatologists (Figure 2). Interestingly, except for vitamin D3 analogues, other topical therapeutics played a minor role. In the Medicare population, vitamin D3 analogues played a similar important role (13.9%; [28]). We analysed calcipotriol prescriptions for mono- or combination preparations subsequently. According to the guideline, monopreparations should not be prescribed over a period of more than two consecutive quarters. In this case, the prescription of combination preparations is recommended [8]. Here, it was shown that combination preparations were predominantly prescribed before and after NHA for over 50% of the study population. A total of 25.9% and 36.0% received monopreparations in more than two quarters prior and after NHA, respectively. However, the very small number of cases must be taken into account. Basically, a study showed that the two-component ointment is effective and well tolerated in the treatment of psoriasis vulgaris, regardless of the age group [35].

Basic therapeutics are rarely prescribed. This may be because they can be bought as non-prescription medicines or over-the-counter products and therefore do not appear as a billed prescription in health insurance data. A reliable statement on the use of basic therapeutics is therefore not possible—especially in the context of the missing formulations, which were not part of the present data set. In our analysis, phototherapy was also rarely used before NHA and did not occur after NHA. Phototherapy comes, above all, along with a logistical challenge in the nursing home setting: the patient must be brought to special centres or practices for phototherapy and needs to have sufficient cognitive ability to tolerate the treatment. Phototherapy is an important component of psoriasis therapy, but studies on the use and tolerability of phototherapy in elderly patients are scarce [27,36]. Apparently, the general practitioner plays an important role as prescribing physician.

### 4.5. Strength and Limitations

A clear strength of this study is that we included people of older age regardless of cognitive status, degree of frailty or access to the research field. This is especially true for people receiving care in the NH setting. The lack of clinical parameters in health insurance data makes it difficult to assess the overall care situation. Statements on the severity of psoriasis are therefore limited. The identification of severe cases of psoriasis based on the use of systemic therapeutics has been successfully applied in many studies [37]. When evaluating and interpreting these data, it must be considered that the medication may also be prescribed based on other clinical pictures. In particular, topical and systemic steroids are intended for a broad the spectrum of indications.

## 5. Conclusions

For the first time, our study shows which drugs for therapy of psoriasis are prescribed in the special life phase of nursing home entry in psoriasis patients of advanced age. Overall, there are only few data on the use and tolerability of current therapeutic options. Topical steroids as well as vitamin D3 analogues (with much less frequency) are the most frequently prescribed topical agents. Systemic therapies play a minor role. There is no considerable change in prescription patterns between before and after NHA, but a mild shift from dermatologists to GPs, indicating a largely retained access to care after admission to nursing homes. Nevertheless, there is much space for improvement with respect to guidelines compliant healthcare. A structured assessment of the skin is crucial, specifically in people with cognitive impairment. The development of an expert standard for ageing skincare and observation is needed to enable adequate care of ageing (psoriatic) skin. In addition, specific treatment guidelines in the geriatric population are needed to optimise the therapeutic approach in this population.

## Figures and Tables

**Table 1:** Psoriasis diagnosis (ICD-10).

ICD-10 Diagnosis	Term
L40	Psoriasis
L40.0	Psoriasis vulgaris
L40.1	Generalized pustular psoriasis
L40.2	Acrodermatitis continua
L40.3	Pustulosis palmaris et plantaris
L40.4	Guttate psoriasis
L40.5	Psoriatic arthritis (PsA)
L40.8	Psoriasis, unspecified
L40.9	Other psoriasis

**Table 2:** Substances indicating a severe form of the disease (approval before 2016).

ATC Information	Active Ingredients
**Biological systemic medication**	
L04AB04	Adalimumab
L04AB02	Infliximab
L04AC05	Ustekinumab
L04AC10	Secukinumab
L04AB01	Etanercept
**Nonbiological systemic medication**	
L04AA32	Apremilast
L01BA01	Methotrexate
M01CX01	Methotrexate
L04AX03	Methotrexate
L04AD01	Ciclosporine
D05BB02	Acitretin
D05BX20	Alkyl esters of fumaric acid
D05BX01	Fumaric acid
D05BX51	Fumaric acid derivatives, combinations

**Table 3:** Psoriasis-relevant systemic therapeutics (approval before 2015) [21].

ATC Information	Active Ingredient
**Biological systemic drugs**	
L04AB04	Adalimumab
L04AB02	Infliximab
L04AC05	Ustekinumab
L04AC10	Secukinumab
L04AB01	Etanercept
**Nonbiological systemic drugs**	
L04AA32	Apremilast
L01BA01	Methotrexate
M01CX01	Methotrexate
L04AX03	Methotrexate
L04AD01	Ciclosporine
D05BB02	Acitretin
D05BX20	Alkyl esters of fumaric acid
D05BX01	Fumaric acid
D05BX51	Fumaric acid derivatives, combinations
D05BA03	Bergapten
D05BA02	Methoxsalen
D05BA01	Trioxysalen
**Systemic steroids**	
H02AB	Glucocorticoids
H02B	Corticosteroids for systemic use, combinations

**Table 4:** Topical therapies according to ATC codes [21].

ATC Information	Active Ingredient
**Tars**	
D05AA	Tars
**Dithranol**	
D05AC01	Dithranol
D05AC51	Dithranol, combinations
**Psoralen**	
D05AD01	Trioxysalen
D05AD02	Methoxsalen
**Vitamin D3 analogues**	
D05AX02	Calcipotriol
D05AX03	Calcitriol
D05AX04	Tacalcitol
D05AX52, D05AX22	Calcipotriol, combinations
**Salicylic acid**	
D05AX56	Salicylic acid, combinations
D02AF	Salicylic acid preparations
**Calcineurin inhibitors**	
D11AH01	Tacrolimus
D11AH02	Pimecrolimus
D11AX14 (until 2010)	Tacrolimus
D11AX15 (until 2010)	Pimecrolimus
**D07A—Corticosteroids, plain**	
D07AA	Corticosteroids, weak (group I)
D07AB	Corticosteroids, moderately potent (group II)
D07AC	Corticosteroids, potent (group III)
D07AD	Corticosteroids, very potent (group IV)
**D07B—Corticosteroids, combinations with antiseptics**	
D07BA	Corticosteroids, weak, combinations with antiseptics
D07BB	Corticosteroids, moderately potent, combinations with antiseptics
D07BC	Corticosteroids, potent, combinations with antiseptics
D07BD	Corticosteroids, very potent, combinations with antiseptics
**D07C—Corticosteroids, combinations with antibiotics**	
D07CA	Corticosteroids, weak, combinations with antibiotics
D07CB	Corticosteroids, moderately potent, combinations with antibiotics
D07CC	Corticosteroids, potent, combinations with antibiotics
D07CD	Corticosteroids, very potent, combinations with antibiotics
**D07X—Corticosteroids, other combinations**	
D07XA	Corticosteroids, weak, other combinations
D07XB	Corticosteroids, moderately potent, other combinations
D07XC	Corticosteroids, potent, other combinations
D07XD	Corticosteroids, very potent, other combinations

**Table 5:** Baseline characteristics of patients with psoriasis in the year before and after entering a nursing home (N = 718) [23].

	12 Months Pre NHA *	12 Months Post NHA
**Mean age at NH entry, years (** **SD)**	83.3 (7.5)	84.3 (7.5)
**Age groups, in years, n (%)**		
65–74	109 (15.2)	
75–84	251 (35.0)	
85+	358 (50.0)	
**Sex, n (%)**		
Male	171 (23.8)	
Female	547 (76.2)	
**Care level, n (%)**		
0/1	456 (63.5)	
2	231 (32.2)	
3	31 (4.3)	
**Severity of disease, n (%)**		
Mild	694 (96.7)	699 (97.4)
Moderate to severe	24 (3.3)	19 (2.6)
**Prevalence of psoriasis-related comorbidities, n (%)**		
Diabetes mellitus	277 (38.6)	258 (35.9)
Obesity	120 (16.7)	82 (11.4)
Hypertension	612 (85.2)	571 (79.5)
Ischemic heart disease	268 (37.3)	226 (31.5)
Osteoporosis	240 (33.4)	213 (29.7)
Depression	293 (40.8)	248 (34.5)
Cataract	214 (29.8)	184 (25.6)
Pruritus	51 (7.1)	64 (8.9)
Dementia	382 (53.2)	441 (61.4)

NH, nursing home; NHA, nursing home admission; SD, standard deviation. * incl. index quarter.

**Table 6:** Top five most prescribed drugs in percentage of all prescriptions in the year prior vs. after nursing home admission (NHA) in patients with psoriasis (N = 718).

	12 Months Pre NHA *			12 Months Post NHA		
	ATC Code	Agent	Frequency, n (%)	ATC Code	Agent	Frequency, n (%)
**1**	N02BB02	Metamizole natrium	1601 (5.7)	N02BB02	Metamizole natrium	2688 (8.1)
**2**	C03CA04	Torasemide	962 (3.4)	A02BC02	Pantoprazole	1332 (4.0)
**3**	A02BC02	Pantoprazole	847 (3.0)	C03CA04	Torasemide	1277 (3.8)
**4**	C09AA05	Ramipril	724 (2.6)	C09AA05	Ramipril	834 (2.5)
**5**	C07AB02	Metoprolol	702 (2.5)	C07AB02	Metoprolol	713 (2.1)

* incl. index quarter.

**Table 7:** General information on drug therapy (N = 718) in people with psoriasis before and after nursing home admission (NHA).

	12 Months Pre NHA *	12 Months Post NHA	*p*-Value
People with psoriasis with at least one drug prescription, n (%)	715 (99.6%)	718 (100%)	0.2482
Number of drug prescriptions, n (mean; SD)	27,936 (39.1; 0.9)	33,301 (46.4; 22.8)	<0.0001
Prescriptions for different agents, n (SD)	14.3 (6.5)	13.2 (5.6)	<0.0001
People with psoriasis with at least one prescription for a psoriasis-related therapy, n (%)	386 (53.8%)	382 (53.2%)	0.7995

SD, standard deviation. * incl. index quarter.

**Table 8:** Systemic therapies in insured people with psoriasis before and after NHA (N = 718).

	12 Months Pre NHA *	12 Months Post NHA	*p*-Value
**Systemic drugs (without steroids), people with psoriasis with at least one prescription, n (%)**	19 (2.6)	15 (2.1)	0.2482
**DDD, n (mean, SD)**	3003.1 (Ø 158.1; ±12.6)	2992.4 (Ø 199.5; ±103.5)	0.9874
**Biological drugs, n (%)**	2 (0.3)	2 (0.3)	-
**DDD (mean, SD)**	542.9 (Ø 271.4; ±101.0)	685.7 (Ø 342.9; ±0.002)	0.3282
**Nonbiological drugs, n (%)**	17 (2.4)	13 (1.8)	0.2482
**DDD (mean, SD)**	2460.3 (Ø 144.7; ±124.2)	2306.7 (Ø 177.4; ±92.6)	0.8230
**Systemic steroids, n (%)**	126 (17.5)	102 (13.8)	0.0097
**DDD (mean, SD)**	22,702.4 (Ø180.2; ±178.8)	21,618.7 (Ø 212.0; ±219.1)	0.6423
**Stratified analysis of systemic steroids**			
**Age in years**, **n (%)**			
65–74	18 (16.5)	14 (12.8)	0.2059
75–84	58 (23.1)	42 (16.7)	0.0077
85+	50 (14.0)	46 (12.8)	0.5271
**Sex**, **n (%)**			
Male	22 (12.9)	15 (8.8)	0.0522
Female	104 (19.0)	87 (15.9)	0.0466
**Level of care**, **n (%)**			
0/1	79 (17.3)	66 (14.5)	0.0687
2	42 (18.2)	31 (13.4)	0.0482
3	5 (16.1)	5 (16.1)	1.000

DDD, defined daily dose; SD, standard deviation; NHA, nursing home admission. * incl. index quarter.

## Data Availability

The datasets analysed during the current study are not publicly available as it concerns health insurance data.
